# Digital Resilience Among Individuals in School Education Settings: A Concept Analysis Based on a Scoping Review

**DOI:** 10.3389/fpsyt.2022.858515

**Published:** 2022-03-31

**Authors:** Haiyan Sun, Changrong Yuan, Qian Qian, Shuzhi He, Qiong Luo

**Affiliations:** ^1^School of Nursing, Jiangsu Vocational College of Medicine, Yancheng, China; ^2^School of Nursing, Fudan University, Shanghai, China

**Keywords:** digital resilience, school education, psychosocial behavior, digital technology, concept analysis, scoping review

## Abstract

**Background:**

Nowadays, in an informational society, digital technologies are present in most areas of life, including school education fields. Students encounter risks or threats during online experiences. Digital resilience helps individuals recognize and manage the risks and threats they come across when they socialize, explore, or work online and plays an important role in the digital technology challenges. However, so far, the concept of digital resilience among individuals in the educational field has not been analyzed in detail.

**Objectives:**

The purpose of this study is to clarify the concept of digital resilience among students in a school education context, describe antecedents and consequences, and suggest a conceptual model for health educators.

**Methods:**

Walker and Avants’ concept analysis method and standards of the scoping review were used to clarify the attributes, antecedents, and consequences from the included articles. A thematic analysis approach of literature was utilized to describe the study findings. No date limitations were applied.

**Results:**

A total of 22 included articles provided data for digital resilience conceptualization. Five defining attributes for the concept were identified as follows: (1) understanding online threats; (2) knowing solutions; (3) learning knowledge and skills; (4) recovering from stress; and (5) moving forward through self-efficacy. Antecedents included digital technology-related threats influenced by individual external and internal factors. Consequences were divided into two categories: behavioral performance and psychosocial functioning.

**Conclusion:**

Based on the results of the concept analysis, a preliminary conceptual model of digital resilience was described as a circular process toward greater performance and function in the form of understanding, knowing, learning, recovering, and moving forward, when facing stressors, challenges, or adversity. The conceptual model of digital resilience can be further tested and may inform the enhancement of digital-specific resilience measures and interventions for students.

## Introduction

The concept of resilience has been receiving increasing interest from various disciplines of study such as psychology, ecology, sociology, education, epidemiology as well as social work trauma studies. Due to the increasing interest in resilience research, resilience has been contextualized at an individual level, family level, community level, national level, and cultural level (1). This study is interested in resilience as an individual’s trait. On an individual level, resilience is generally understood as the ability to cope with the challenges, stresses, and adversities in life (2).

Since the era of big data, researchers have been studying the impact of digital technologies on health outcomes. Undeniably, digital technologies could promote human health development in many ways (3). However, people may encounter risks when using digital technologies and it is neither possible nor desirable to shield them entirely from these risks. Consequently, the increased use of digital technology has led to a spike in the rates of webinar fatigue, technology-use anxiety, and digital burnout, due to the difficulty of maintaining clear work–life boundaries (4). Interestingly, the majority of people adopt a positive attitude and behavior to cope and hope to reduce the occurrence of negative outcomes. Obviously, individuals witnessed the digital revolution and showed new forms of resilience in the digital context.

This new form of resilience in the digital space is known as “digital resilience.” The concept of digital resilience was originally applied within information technology fields. Early studies on digital resilience focused on organizations’ technologies and enhancing their ability to adapt and safeguard their system’s normal operation when there are informational technology challenges (5, 6). Casalino et al. (7) depicted digital resilience as a constellation of strategies, practices, policies, and programs that safeguard a society’s ability to maintain, change, and recover digital capabilities and withstand digital crises and shocks in the organizational level. In technical orientation, digital resilience is described as the technical capabilities of systems and infrastructure to show that they are running continuously after being attacked (8). For communities or societies, data and tools should be freely accessible, interchangeable, operational, of high quality, and up to date (8). Digital resilience in academia is mostly talked about in terms of cybersecurity. Some definitions take a behavioral orientation as they refer to digital resilience as technology adoption process against attacks (9, 10). Consequently, most of the proposed conceptual frameworks address the technologies used in achieving digital resilience instead of the human factor. As of now, the phenomenon of individuals’ digital resilience is missing a clear conceptualization. In this study, the core element is human individuals in the psychological and behavioral aspects.

There are different conceptual constructions exploring or proposing frameworks to evaluate, improve, or measure digital resilience among individuals. Initially, the concept of resilience was developed in a socio-technical aspect of digital resilience during disruptive life events at an individual level. For instance, Hua et al. (11) proposed an economic resilient behavior model to study individual psychological resilience in the context of cyberterrorist attacks on financial systems. Equivalently, Budak et al. (12) conducted a study that customers responded to online privacy violations and the behavioral implications of the stressful occurrence. Later, this concept was widely expanded into the human psychobehavioral aspect. Eri et al. (13) extended the theory of social–ecological resilience in the digital technology context and defined digital resilience as the ability of learners to overcome technological difficulties and persist with online learning as they adapt to the changing trends in higher education. However, digital resilience firstly occurred in school education settings due to the massive open online courses and open-access publishing. The authors proposed a framework of digital resilience describing an institution’s ability to adapt to digital challenges (14). Since the beginning of 2020, the coronavirus disease 2019 (COVID-19) worldwide pandemic has accelerated the innovation of technology in every field, and the levels of digital use have significantly increased through the pandemic. Consequently, employees were expected to work online, students were expected to study remotely, patients at one point were expected to attend online consultations, and the public was expected to conduct daily errands online due to sudden lockdowns (15). The lockdown and social distancing measures led to a significant change in the modes of work and learning. While the benefits of using technology to maintain day-to-day norms are clear, spending more time online can also increase the risk of encountering issues. This issue thus leads to a concept of how aware the students are of their digital resilience.

The risks of problematic digital behavior like digital burnout, mental health distress caused by digital failures, or unhealthy interactions on social media such as cyberbullying and cybercrime are a huge pressure for individuals. There is a need to enable individuals to build digital resilience and understand mediating psychological concerns to enhance digital resilience (16). In the last few years and above all in the last 3 years, there have been multiple attempts by scholars to define digital resilience. Some definitions agree that digital resilience is the technical capabilities of systems and infrastructure to show resilience (5, 6, 8, 17). Other definitions take a behavioral change as they refer to digital resilience as technology adoption (4, 12, 16, 18), yet the definition of digital resilience is either indistinct or inconsistent in different studies. In addition, in many cases, there is a general conflation between “digital literacy” and “digital resilience” (19). Tran et al. (20) proved a positive relationship between digital resilience and digital literacy. Digital literacy as a necessary skill of future digital citizens was correlated with greater resilience levels. Budak et al. (12) found that the digital literacy set needed to achieve digital resilience was not generic and depends on the context of the stressful event, but it had been found as a factor influencing individual resilience online. Therefore, digital literacy is generally concerned with the effective and ethical understanding and use of digital technologies, whereas digital resilience is related to the capacities of accessing, using, understanding, and spreading effective digital sources and common manipulative techniques, in particular, behavioral and attitudinal change aspects.

From the abovementioned studies, the definition of digital resilience remains to be heterogeneous across different studies. Furthermore, in the social psychological behavior of digital resilience among different populations, some studies even gave controversial results (13, 21). In addition, some concepts or phrases exist that have similar meanings to that of digital resilience (e.g., information literacy or digital literacy), which are likely to cause confusion. To address these knowledge gaps, we analyze the concept of digital resilience to gain an in-depth comprehension of its components and support the development of theories. Therefore, our aim is to identify the concept of digital resilience, including the underlying processes, and more specifically for online learning students in school education.

## Methods

Concept analysis is used to examine the basic elements of a concept to investigate its structure and function (22). This study draws on a recognized concept analysis method to conceptualize the digital resilience of students in the context of school education (22). There is an eight-step process for concept analysis, which is shown in Table 1.

**TABLE 1:** Eight steps for concept analysis as interpreted from Walker and Avant.

Order	Specific content
Step 1	Identify and select concept
Step 2	Clarify the purpose of analysis
Step 3	Identify the uses of concept
Step 4	Determine the defining attributes
Step 5	Construct a model case
Step 6	Construct additional cases
Step 7	Identify antecedents and consequences
Step 8	Define empirical referents

### Concept Selection

As a concept, digital resilience is mostly talked about in terms of cybersecurity but it is still unclear at an individual social psychological behavior level. A concept analysis is used based on Walker and Avant (22) because this method is applicable that is prevalent in practice and has been used across disciplines.

### Purpose of Analysis

Concepts describe the phenomena of interest, and a better understanding and application of the concept is able to promote health and wellbeing in research. The strength of a concept depends on it being clearly defined (22). However, as a concept, digital resilience is much talked about but ill-defined, so the key step is to agree on an unambiguous definition of what it actually means in a special context. Therefore, the purpose of this concept analysis is to validate the meaning of digital resilience, developing an operational definition of the concept within the context of higher education.

### Literature Search Strategy

As we aimed to map the literature about digital resilience involving individuals, we decided on a scoping review methodology. Such a review is systematic and focuses on central concepts and an overview of the current state of research (23). A systematic search was conducted in electronic databases including Web of Science, PubMed, CINAHL, EBSCO, PsycINFO, Wiley, Scopus, and ProQuest on November 20, 2021. We used the keywords “digital resilience” OR “digitally resilient” OR “digitally resilient” OR “digital Resiliency” OR “online resilience” to retrieve relevant literature and bibliography. The Preferred Reporting Items for Systematic reviews and Meta-Analyses extension for Scoping Reviews (PRISMA-ScR) checklist was utilized to ensure completeness in the scoping review (24). This process therefore systematically, transparently, and comprehensively identifies, selects, analyzes, and synthesizes the body of literature where the conceptual expression of digital resilience is found to identify the attributes, antecedents, consequences, references, surrogate, and similar concepts and contextual variations. No date limitations were applied to the search strategy. All the studies published in English focused on digital resilience in individuals. We included peer-reviewed original publications, reviews, guidelines, and gray literature.

### Article Selection

**FIGURE 1:**
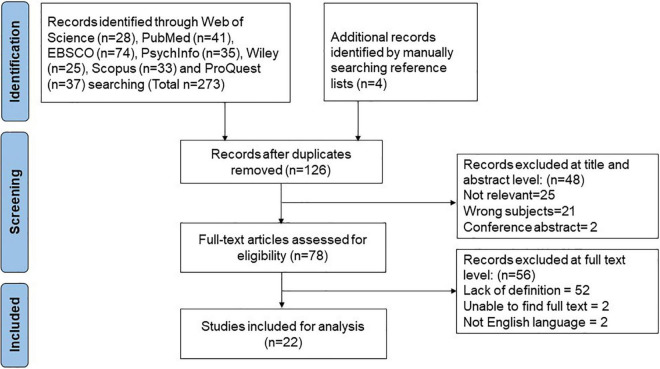
Flow chart of study selection process.

The selection process was carried out by two independent assessors (HS and QQ). This selection was validated by a third researcher (SH). The inclusion criteria consisted of the concept of at least one of the following items: definitions, attributes, antecedents, consequences, and/or methods for digital resilience measurement. Conference abstracts and studies that examined digital resilience beyond or below the level of individuals (organization, institution, community, and system) were excluded from the analysis. The study selection process was presented in Figure 1. A total of 277 results were found, and 151 studies were excluded after removing duplicates. Based on title and abstract assessments, 48 studies were excluded because of irrelevant researches, book reviews, letters to the editor, and studies published in other languages. Afterward, 56 studies were excluded in terms of the inclusion and exclusion criteria by screening the full text. Finally, 22 articles and records were included as they were particularly relevant to this concept analysis.

### Data Analysis

**FIGURE 2:**
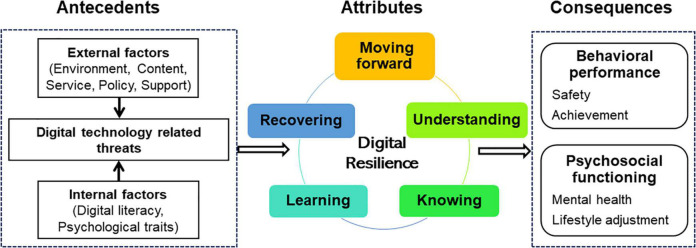
Preliminary conceptual model of digital resilience.

To analyze data, we followed a two-phase approach. In the first phase, we systematically extracted basic information from each article, including authors, the year, countries, study method, population, and definition as shown in Table 2. In the second phase, the thematic analysis approach was used to identify attributes, antecedents, and consequences. Data were explored for contexts for meaning based on identifying the relevant characteristics of digital resilience. We categorized common themes through multiple iterations of the included studies of the articles in Table 3. Ultimately, the thematic network of the digital resilience concept’s attributes, antecedents, and consequents was built in Figure 2.

**TABLE 2:** Articles included for the final analysis.

No.	Authors/Year	Study population	Country	Research design	Definition in digital resilience
1	(18)	Children	India	Literature review	A way of coping with the digital challenges (e.g., MOOCs, open-access publishing, risk), or resilience as the final aim of a project by implementing digital methods (e.g., digital storytelling, social networks)
2	(25)	Adolescents living in out-of-home	United Kingdom	Longitudinal qualitative study	An orientation that recognizes digital vulnerabilities and seeks to empower the susceptible individuals to navigate toxic elements in the context of supportive relationships
3	(26)	The youth	South Africa	Qualitative study	The ability to acquire new digital skills that can help an individual to navigate increasingly digitally oriented, dynamic societies
4	(27)	12–17-year-old middle and high school students	United States	Quantitative study	The capacity to spring back, rebound, successfully adapt in the face of adversity, and develop social and academic competence despite exposure to severe stress or simply the stress of today’s world
5	(19)	Vocational college students	Netherlands	Mixed-method research	The responsible, safe, and active participation in online communities having the capacities of critical thinking, media literacy, social behaviors online, peer safeguarding, and the law online
6	(4)	Tertiary-level students	Australian	Qualitative study	An individual student’s psychological capacity to remain functional by absorbing, recovering from, adapting to and learning from adversities stemming from the use of digital technology in the tertiary educational context
7	(28)	Children and young people	United Kingdom	Scoping review	A dynamic personality asset that grows from digital activation through engaging with appropriate opportunities and challenges online, rather than through avoidance and safety behaviors
8	(29)	Children and young people	United Kingdom	Qualitative study	The responsible, safe, and active participation in online communities
9	(30)	Citizens	Thailand	Field study	Thai citizens are able to utilize digital technologies to help themselves withstand the COVID-19 pandemic
10	(20)	Secondary students	Vietnam	Cross-sectional study	Students could protect themselves and others from online risks and recover and learn from risky situations
11	(10)	Learners, instructors, policymakers	United States	Scoping review	The ability to adopt new digital technology solutions quickly and seamlessly in order to recover, rebound, and move forward if things go wrong
12	(31)	University students	United Kingdom	Literature review	Students develop an awareness to cope with adversity and challenges from technology and develop positive strategies for their technology use
13	(32)	First-year student teachers	United Kingdom	Cross-sectional study	Student teachers’ ability to avoid disturbances caused by or the distractions of digital technology during their studies
14	(33)	Children	Thailand	Literature review	Digital resilience is a necessary skill for digital citizens that they can learn to deal with online violence using their coping capacities
15	(34)	Syrian refugees	Netherlands	Qualitative study	Engaging in digital media to cope, escape, feel stronger, fight back, find, and foster community in a new environment after the systemic violence and historic trauma
16	(13)	Tertiary students	Australia	Mixed-method approach	Students’ tech-savviness and preparedness to adapt to different digital environments as they pursue higher education
17	(16)	All individuals	India	Literature review	A new concept that refers to the learning, recovery, and bouncing-back process after having negative or adverse experiences online
18	(35)	Remote working employees	United Kingdom	Cross-sectional study	People have e-skills, trust building, self-care, remote social, and remote emotional self-efficacy
19	(9)	Residents aged 18 years or older	Qatar	Quantitative research	Refers to an individual being cognitively well placed to adopt new technologies and accept digital transformation as a way to bounce back from disruptive events
20	(21)	Children and adolescents	United Kingdom	Qualitative study	The ability to be confident online or having a reasonable refusal in a situation or to de-escalate negative online communication
21	(36)	Translation students	Poland	Qualitative study	Be understood as a way of coping with digital challenges grounded in the concept of learner autonomy
22	(12)	Consumers	Croatia	Literature review	Consumers can change their attitudes and behaviors to withstand, recover from, and reorganize after an online privacy breach

*UKCIS, UK Council for Internet Safety.*

**TABLE 3:** Attributes, antecedents, and consequences of digital resilience in the included studies.

No.	Authors/Year	Descriptions in attributes	Descriptions in antecedents	Descriptions in consequences
1	(18)	Learning digital methods	Environment: Challenges in medical curricular changes	Achievement: Lifelong learning
2	(25)	Understanding digital media Knowing risk management Moving forward with self-empowerment	Environment: Digital media usage Policy: Top–down cyber-safeguarding risk-management policies	Safety: Keeping safe online
3	(26)	Learning digital skills Recovering in reaction and behavior	Service: Digital infrastructure, access to local digital Support: Family Digital literacy: Skills and knowledge	1. Achievement: Improving academic achievement, and increasing self-efficacy, leadership, and empathy skills 2. Lifestyle adjustment: Long-term behavioral change
4	(27)	Understanding stress Recovering formal life Moving forward by social and academic competence	N/A	Safety: Preventing bullying and cyberbullying victimization
5	(19)	Knowing with critical thinking Learning media literacy and social behaviors online, peer safeguarding, and the law online	Digital technology-related threats and service: Extremist grooming and exploitation online Environment: The Internet and social media Digital literacy: Knowledge and skills Psychological trait: Attitudes and behaviors	Safety: Using social media effectively, safely, and constructively as effective digital citizens
6	(4)	Self-learning reactively and proactively Recovering through reactive self-efficacy and proactive self-efficacy	Psychological trait: Self-motivation toward study Support: Peer influence, university support	Achievement: Helping students achieve positive learning outcomes
7	(28)	Understanding when you are at risk Knowing what to do to seek help Learning from experiences Having appropriate support to recover	Psychological trait: Mental and cognitive health issues, vulnerabilities External factors: Environment, content, service, policy	N/A
8	(29)	Knowing using critical thinking Learning in digital literacy	Environment and content: Innovative social media simulations	Safety: Using online technology safely and appropriately
9	(30)	Learning how to optimize the use of technology Recovering life in overstepping space constraints	Environment: Information and communication technologies, the rise of information society	Mental health: Helping citizens’ mental health during lockdown or mobility restriction
10	(20)	Learning a necessary skill can use digital tools Recovery in handling online risks	Digital literacy: Skills and knowledge Support: Society, family Psychological trait: Girls are more likely to obtain digital resilience than boys	Achievement: Helping students to achieve a better outcome from online learning methods
11	(10)	Recovery from the crisis Moving forward into a new reality	Policy: COVID-19 pandemic lockdown	Lifestyle adjustment: Delivering education Achievement: Promoting lifelong learning
12	(31)	Knowing balanced technology use Moving forward the transition to university life	Digital technology-related challenges and environment: Technology use transferring to a virtual context	Mental health and safety: Have the skills and confidence to conduct themselves safely in an online world
13	(32)	Understanding the importance of engaging responsibly with the digital world Recovery from dissolving the boundaries between online and off	Digital literacy Service: Online activity Content: Free information resources	Lifestyle adjustment: More time spent on individual studies Achievement: Benefit learning and working
14	(33)	Understanding digital environment Learning coping skills, problem-solving skills, and emotional literacy	Digital literacy Psychological traits: Self-esteem, psychological difficulties Support: Parental support, peer norms, school educational support Service: Kind of sites, apps, etc	Mental health: Cognitive responsibilities
15	(34)	Knowing methods in coping adversity Recovery in feeling stronger and fighting back Moving forward in finding and fostering new integration	Environment and service: Information and communication technology Social support	Health promotion and wellbeing safety: Identity management
16	(13)	Understanding technological difficulties Learning digital competence and skills Recovering with psychological resilience Moving forward with confidence	Digital technology-related challenges: Digital learning stress Environment: Digital technology innovation Service and policy: Learning environments changing Digital literacy: Prior knowledge and experience with digital technology	Lifestyle adjustment: Digital transformation and the new normal of higher education Mental health: Students’ wellbeing, mitigating social isolation Achievement: Pursuing higher education
17	(16)	Understanding the awareness of online etiquette and digital deviance Knowing behavioral inhibition Learning digital hygiene skills Recovery in emotional regulation	Digital technology-related threats: Digital burnout, webinar fatigue, cyberbullying, cybercrime, doom surfing doom scrolling, digital failures Service: Awareness and impairing education about healthy and deviant digital use Support: Family Digital literacy: Digital hygiene skills, behavioral inhibition Psychological traits: Emotional regulation, confidence, self-esteem, flexibility, coping strategies, etc	Mental health: Cyber wellbeing Lifestyle adjustment: Reducing the adverse effects of excessive online usage
18	(35)	Learning knowledge and skills Recovery in attitudes and behaviors	Environment: Rapid advancements in the technology of remote working practices	Achievement: Promoting sustainable, productive, engaging, and healthy remote working and delivering the best personal and work outcomes
19	(9)	Recovering after hardship Moving forward when faced with challenges	Psychological trait: Self-efficacy Digital literacy: Skills and knowledge Support: family, community capital	Lifestyle adjustment: Raising the level of digital readiness to the workforce
20	(21)	Recovering with a kind of psychosocial skill	Risks and threats in digital technologies	Lifestyle adjustment: Controlling online behaviors Safety: Protecting internet addiction and risk behaviors
21	(36)	Learning knowledge and skills	Environments: Distance learning—new spaces for learning in the new training environments outside the classroom	Lifestyle adjustment: Improving overall learner autonomy Mental health: Gaining more confidence in learning
22	(12)	Moving forward the adaptive capacity in behavior and attitudes	Digital technology-related threats: Online privacy violation Digital literacy: Attitudes toward Internet usage Support: Social support, family relationships, peers	N/A

*N/A means that the included articles did not provide the content.*

## Results

### Studies Included

Based on the above inclusion and exclusion criteria, 22 studies were retained for the final analysis, including 10 qualitative studies, 5 quantitative studies, 2 mixed-method studies, 5 various reviews, and 1 field study. The dates of publication ranged from 2014 to 2021. The studies were conducted in 11 countries, which were mainly distributed in Europe, Asia, and North America. The studies including the details are presented in Table 2.

### Uses of the Concept

The third step is to identify the various utilizations of the concept by consulting different resources such as dictionaries, encyclopedias, and research databases (22). The aim of this step is to scope the possible uses and definitions of digital resilience.

The Oxford English Dictionary (2018) defines “digital” as “using a system of receiving and sending information as a series of the numbers one and zero, showing that an electronic signal is there or is not there” or “connected with the use of computer technology, especially the internet” or “showing information in numerical digits.” Indeed, we live in the Age of Big Data because digital technologies have become an inevitable part of our daily lives by changing how humans interact. There are some risks or threats to digital technologies, so it is neither possible nor desirable to be entirely away from risks.

Resilience is a very commonly used term within multiple disciplines. *Merriam-Webster Dictionary* defines resilience as “the ability to recover from or adjust easily to change or misfortune” (37). The *Oxford English Dictionary* defines “resilience” as (1) “the capacity to recover quickly from difficulties; toughness” or (2) “the ability of a substance or object to return to its original shape; elasticity” (38). *Mosby’s Dictionary of Medicine, Psychology and Health Professions* defines resilience as “the ability of a body to return to its original form after being stretched or compressed” [(39), p. 1,606].

Different disciplines had divergent understandings of the concept of resilience. In physics and engineering sciences, resilience is conceptualized as material strength and the ability of the material to return to its original shape after physical strain (40). From an ecological perspective, resilience is the ability of an ecosystem to absorb disturbances and reorganize while undergoing change so as to continue to retain essentially the same function, structure, feedback, and therefore identity (41). In the biology field, resilience is conceptualized as the ability of an organism to regenerate in a dynamic process (42). Resilience is also applied in business with corporate trends, money, production, and the stock market with their ability to manage crises and recover from economic or market downturns (43). In the social sciences, such as psychology, social work, and nursing, the meaning of resilience shifted to a process of growth and adaptation, not only a state of bouncing back after experiencing adversity or challenges (44). Recently, research has expanded to the cybersecurity field. In these studies, resilience is linked to the ability of organizations to continue to carry out their mission by anticipating and adapting to cyber threats and other relevant attacks (5).

The above-mentioned studies focused on contextualization in different settings and disciplines, and lately, it also has been introduced into the digital field. Although the context of the concept may change, a common theme is that resilience is the ability to recover and return from threats or adversities. Some state that resilience can be understood as a stable personality trait, while some define resilience as a dynamic process that changes over time (1). Considering the popularity of the concept within various disciplines, a wide range of definitions is available.

Given the digital revolution witnessed in this epoch, individuals showed new forms of resilience through technical innovations. To narrow the search for this concept analysis, the focus is on individual resilience and more specifically on digital resilience. Previous researches about digital resilience not only concerned the community, organization, or national level but also perceived resilience at the individual social psychological behavior level (45). Digital technological innovation cannot be defined as adversity, but it can certainly be described as a major life challenge. In the first study on digital resilience in higher education, the concept was defined as the “utilization of technology to change practices in order to adapt to new circumstances while retaining the underlying function of the practices” (14). Then, the construct of digital resilience is rising in the last decade, especially during the coronavirus disease 2019 (COVID-19) worldwide pandemic. There is a two-fold link between resilience and digital tools: digital tools promote resilience in education and health education; resilience is a means to face digital challenges (18). It is necessary to make a clear definition of digital resilience and its possible antecedents and consequences in the field of education for students’ development. Furthermore, its uses also help to extend implications in other populations.

### Defining Attributes

Determining the defining attributes of a concept offers an assembly of characteristics most often associated with the concept and improved discernment of the concept. The following attributes were compiled after doing an extensive screening about digital resilience in the included studies (*n* = 22). In a consultation with the research team, there are five connected elements that were developed as digital resilience attributes in Table 3. These five attributes are the process of dynamic circulation. Therefore, digital resilience is understanding when you may be at risk online, knowing what to do to seek help, learning knowledge and skills from experiences, being able to recover from appropriate support, and moving forward through self-efficacy in challenges.

#### Understanding Online Threats

Understanding online threats is the first term to be used throughout digital resilience literature (16, 25, 33). Individuals recognize the risks or threats online and can make informed decisions about the digital space that they are in. Diverse students perceive different risks or threats in digital learning environments like problematic online behaviors, cyberbullying, and webinar failures. For example, webinar fatigue makes students feel pressured to attend online meetings and sessions excessively, due to peer pressure, the perceived stress to upskill, and anxiety in the “fear of missing out” (16). Moreover, students may have unhealthy interactions on social media that can lead to deviant behaviors such as cyberbullying and cybercrime (16). In addition, the excessive time spent on digital platforms is a big risk that can cause significant mental health distress to students, which can impact an individual’s lifestyle such as their sleep schedule or appetite (28). Several studies also showed that understanding unhealthy online threats is an important start in building digital resilience (4, 16, 26). Users were encouraged to report harm when using online technologies, which was a style of proactive self-efficacy to reflect their capability to discern risks during online experiences. Therefore, having an awareness of online challenges is necessary for digital resilience.

#### Knowing Solutions

Knowing solutions is a way of critical thinking so as to consider the pros and cons even during challenging situations (19, 25, 28, 29, 33). In other words, individuals know what to do to seek help from a range of approaches and their sources. Knowing the resources about how to manage and act on their behaviors and attitudes by using critical thinking is an imperative attribute for enhancing digital resilience in the school education context. Reynolds and Parker (19) thought that digital resilience is an educational intervention designed to increase the resilience of young people to hate and extremism online. Students were taught to know what opportunities, resources, and skills may be useful to cope in a stressful, disadvantaged, or traumatic situation. In addition, knowing what to do to seek help from family and social support is also conductive to promote critical thinking abilities in their real lives (28). Hammond and Cooper (25) considered digital resilience as an orientation that recognizes digital vulnerabilities and seeks to empower the susceptible individuals to navigate toxic elements in the context of supportive relationships. A healthy support system means making sure that the individual knows when to reach out for support and is made aware of the possible educators or organizations that can help with negative digital experiences (25).

#### Learning Knowledge and Skills

The third digital resilience attribute is learning knowledge and skills including learning how to recognize and manage risk and learn from difficult experiences (19, 20, 25, 28, 29, 35). If individuals have digital skills and knowledge, they can deal with unfamiliar technological circumstances. Learning knowledge and skills does not mean that people should have an expertise in informational technology. A higher level of expertise not only allows better utilization of technology during disruptive life events; it also results in familiarization, which should lead to a reduction in technology anxiety and widen the usage of the technology (9). Individuals learn knowledge and skills from their experience and are able to adapt their future choices where possible (19). In the United Kingdom, some colleges or universities equipped students with colorful knowledge, skills, and strategies and also instructed them to recognize when to access help and support and where to find it (25). Further, resilient students in the digital world are more likely to initiate learning novelties or proactively interact with the environment, not just passively waiting for assistance. Thus, students could protect themselves from online risks and learn from risky situations depending on their level of digital literacy (20).

#### Recovering From Stress

Recovery was selected as one attribute to show the process of adaptation. Individuals can recover when things go wrong online by receiving the appropriate level of support. It is seen as a natural and instinctive characteristic built in all humans through adaptation and evolution. Both Sharma et al. (16) and Al-Abdulghani et al. (46) describe that digital resilience is returning to a pre-event level of functioning (recovery process) after having negative or adverse experiences online. They believed that an individual is cognitively well placed to adopt new technologies and accept digital transformation as a way to rebound from disruptive events. Meanwhile, Eri et al. (13) declared digital resilience as an individual’s psychological capacity to remain functional by recovering from, adapting to, and learning from adversities stemming from the use of digital technology in the tertiary educational context. Obviously, recovery is a vital part of individual development in adopting new digital technology. It is an adaptive capacity for the individuals who adjust based on the immediate situation, knowing past conditions, or predicting future ones. People are also encouraged to seek methods to recover from the events when they suffered severe harm (28). Therefore, recovery represents that an individual bounces back to his/her normal activity as it was before a stressful event happened.

#### Moving Forward Through Self-Efficacy

An important yet often-overlooked consideration when conceptualizing digital resilience is moving forward in the face of new digital technology challenges. Moving forward through self-efficacy is defined as moving on with the belief in the capability to overcome difficulties in the context of supportive relationships (25), and these individuals have the confidence or assertiveness to deal with online challenges successfully in the future (18, 31). By building the aforementioned four attributes of digital resilience, they can help to empower the particularly vulnerable group to keep themselves safer online. Bhagat and Kim (10) described that the trajectories of digital resilience did not stay at the same functional level. It involved bouncing forward into a new reality. Self-efficacy is described as a robust stress-resistance resource after coping to challenges or having the confidence to conduct themselves safely in a digital world. In a study conducted by Rabbanee et al. (4), self-efficacy was found as a significant support to the positive aspects of the use of digital technology in the tertiary educational context. In this digital age, digitally resilient students are also those who have the abilities to avoid disturbances caused by or the distractions of the digital learning environments during their studies, which helps students achieve learning effectiveness (32). Moving forward through self-efficacy was selected as an attribute to reflect the broad characteristics of being well-adjusted in the population (35). It means that an individual is not in the same state as before, and they are thriving at the bounce-back-better level.

### Cases of Digital Resilience

Based on Walker and Avant (22), cases can help illuminate and clarify concepts. Therefore, the fifth and sixth steps involved the development of four types of cases, which were generated from the qualitative data of included studies and the authors’ experience.

#### Model Case

A model case contains all the necessary attributes of a concept (22). Jack is a 20-year-old young university student who participates in a large number of online courses and plays online games every day during COVID-19. He had some negative emotions such as anxiety, worry, irritability, and aggression because of his excessive reliance on online technology. He understands that it adversely affects interpersonal relationships with family members and friends and produces issues of academic stress in the feeling of “fear of missing out” (understanding online threats). He knows that engaging in excessive digital interactions causes work–life imbalance, so he struggles with online fatigue and screen exhaustion. With encouragement from the teachers, Jack attends a series of curricula of student psychoeducation from a healthy school support network (knowing solutions). This helps Jack to learn many problem-solving skills and digital hygiene knowledge including identifying cyberbullying, making plans to attend webinars, and protecting information security (learning knowledge and skills). After that, he gives up attending a lot of online lectures and meaningless online interactions. Jack becomes more self-disciplined to manage study–life transformation in a virtual world (recovery from online stress). Now, to keep this healthy lifestyle, he believes that he is tech-savvy and prepared to adapt to different digital environments as he pursues higher education (moving forward through self-efficacy).

#### Borderline Case

A borderline case is an instance of the concept containing some but not all of the attributes of the concept (22). Selina is an 18-year-old freshman, and she can surf the Internet at any time and any place. She is recommended to use a lot of social software online from classmates and advertisements. These online platforms often send unhealthy messages and pictures to users. Selina is horrified when catching sight of these contents and feels the impact this may have on her health at first. She refuses to join these cyberspace interactions and attempts to seek alternative options to escape them. However, she does not report these cybercrimes to others and does not seek consultation regarding how the practices and behaviors in the digital world are. This leads to the reduction of productivity and satisfaction in her daily learning, although she is mentally able to adapt to the digital environment.

#### Related Case

A related case is an example that is related to digital resilience but does not encompass any defining attributes (22). Allen is a 19-year-old student who is pressured to attend meetings, webinars, and online sessions excessively. She finds that her entertainment, hobbies, work, and even academic study are all from digital sources online. She does not recognize that it is a kind of information overload by the media and news, along with increased stress every day. She conducts some behavioral inhibition to control her excessive use of digital technology.

#### Contrary Case

A contrary case is an example of a concept in which none of the attributes are present (22). Mike is a 21-year-old student in a vocational college. He joins some online courses according to the study requirements during the COVID-19 lockdown, and he spends the rest of the time on various online platforms. When he returns to an offline class 1 day, he feels that he is not the same person as before, when he can attend a face-to-face interaction or classroom discussion, but rather “a man in the cyberworld.” Due to the negative impact of excessive online use and dependence, he always shows lower self-esteem and irritability in learning activities. His classmates also find him abnormality in the behavior and psychology and talk less to him or directly ignore him as they do not know what to say. He is frustrated as his previous image of self-confidence is completely shattered by living in a virtual world. He feels that digital technology controls him, and he has no control of his study and life anymore. As a result, he becomes more addicted to online use and has difficulty maintaining clear work-life boundaries.

### Antecedents and Consequences

According to Walker and Avant (22), identifying antecedents and consequences helps us to have a deeper understanding of a concept within a social context. Antecedents are the events that take place prior to the occurrence of digital resilience, and consequences are those that occur as an outcome of the appearance of the concept (22). Table 3 gives an illustration of the antecedents, attributes, and consequences of digital resilience among individuals.

#### Antecedents

Three antecedents of digital resilience were identified in this analysis: Digital technology-related threat and individual external factors and internal factors.

Firstly, adversity or threat events are common terms used to describe the antecedents of resilience. In the context of digital technology, the adversities are related to the use of digital technology such as cyberbullying (21), webinar fatigue (16), deviant use of technology (16, 19), excessive use of online technology (16, 32), digital burnout or failure (16, 46), and the challenges of technology innovation in a virtual context (18, 36). These threats may be triggered by different external and internal factors and may place an individual at risk of maladaptation.

Secondly, external factors have already existed during the use of digital technologies. The examples of external factors include environment, content, service, policy, and support (4, 28, 34). Environment includes any access point to the internet from private to public spaces, which makes people exposed to stressors such as distance learning and training environments (13). Content includes entertainment and educational content, the terms of service, and any messaging about the use of digital technologies that provide or deliver to the users and may be unhelpful or useless, or even harmful (32). Service contains devices, platforms, apps, games, and websites that may bring many possibilities of changes, challenges, and stressors (16). Policy includes local, national, and institutional policies such as top–down cyber-safeguarding risk-management policies (25). Support includes family, school, and society support networks, and people with support are more resilient and are able to better cope with stress and adversity in their life (33). Students supported by their family, friends, peers, and teachers are more likely to deal with adversities and develop a psychological capacity of resilience.

Thirdly, it is important that the individual internal factors also play a role in facing challenges to activate the resiliency attributes. The internal factors encompassing digital literacy mainly include basic technical knowledge and skills and the psychological traits of coping, which can help individuals build resilience and remedy the impact of adversity or disruptive events. Digital literacy can be used to evaluate information reflecting their capabilities to manage their life and work/learning tasks using digital technologies (19). In addition, individuals need psychological resources to develop resilience under the threat of the online and offline world, including self-control (32), self-reflection (33, 46), self-confidence (13), self-efficacy (12, 46), and self-esteem (33) as positive factors of psychological traits. From the individual viewpoint, digital resilience is understood as a proactively interactive concept dealing with the aggregation of individuals’ severe risk experiences with different relatively favorable psychological resources.

### Consequences

#### Behavioral Performance

Under the influence of digital resilience, the behavioral patterns of students will develop into a positive performance, which explains the impact of digital resilience on good long-term learning behavior. First, digital resilience brings safety, which is an important healthy behavior, and students think that they consider it necessary to surf the web or use online platforms. That is because students have the skills and confidence to conduct themselves safely in an online world (31). Students with responsible, safe, and active participation in an online environment allow them to use social media effectively, safely, and constructively (19, 29). Second, digital resilience encourages students to get a better achievement and explore new online challenges into the learning process. Digitally resilient students sustain motivation, achievement, and performance in their studies while being able to overcome and deal with stressful events (13). Those students with a habit to control online behaviors could be protected against internet addiction and more time spent on individual studies’ development, which benefits learning and working and improves academic achievement (21). Hence, in the presence of digital resilience, students will gradually increase the long-term behavioral change, especially in the field of lifelong learning.

#### Psychosocial Functioning

Students with access to digital resiliency attributes at the time of stress or threat during the use of technology period have more chances to deal with these stressors in a positive way (26, 34). Multiple studies showed that digital resilience had various positive impacts on psychological and social functioning. For psychological functioning, digital resilience may assist students to maintain or promote positive emotions, which better enable them to adjust in the midst of negative emotions or even improve individuals’ cyber wellbeing after such as recognizing what is right in their behavior (16). Especially, digital resilience could help promote individuals’ mental health during the COVID-19 lockdown or mobility restriction (13, 30, 35). With respect to social functioning, research shows that digital resilience is positively correlated with a lifestyle adjustment. People with high levels of digital resilience may manage to adapt to challenging situations and strive to go back to or maintain their previous social life (19). In other words, individuals owing digital resilience show anti-interference ability when they encounter various technical risks or opportunities. Taking COVID-19 as an example, the nature of digital resilience is conducive to mitigate mental fatigue from facing social and academic isolation (13). Those with lower resilience and less adaptation may keep an unhealthy lifestyle paradigm, resulting in decreased anti-interference when encountering various technical risks or opportunities (32).

### Empirical Referents

The final step of the concept analysis is empirical referents for the operational definitions of concept variables (22). To date, no specific instruments of digital resilience have been identified to measure the existence or attributes of the concept. Currently, there are several empirical instruments available to recognize or measure the occurrence of resilience for children, youth, young adults, or older adults. Windle et al. (47) found there are 19 resilience scales commonly used and recommended that the Connor–Davidson Resilience Scale, Resilience Scale for Adults, and Brief Resilience Scale have the best psychometric ratings. However, digital resilience has specific features that differ from resilience in general. To our knowledge, not one widely used tool is fit for scaling digital resilience. Although some authors used self-designed subjective instruments (9, 19, 20, 26, 28) to measure digital resilience in either behavior or attitudes, they did not assess the validity and reliability. In the future, it might be appropriate to measure the defining attributes as separate and additional constructs and be useful to measure not only behavior but attitudes as well.

### Conceptualization of Digital Resilience

Based on the analysis of the defining attributes, antecedents, and consequences of digital resilience, we propose a final, clarified, and refined definition in school education settings. Digital resilience is the capacity and dynamic cycle process of an individual to change their behavioral performance and psychological functioning through understanding the risk, knowing approaches, learning knowledge and skills, recovering from stress, and moving forward when facing various digital technology-related threats within the school education settings. A preliminary conceptual model covering the relationship between attributes, antecedents, and consequences of digital resilience was established based on this conceptualization (Figure 2).

## Discussion

This concept analysis of individual digital resilience in school education applies the Walker and Avant concept analysis method. The aim of this study was to generate a clarified definition of digital resilience for school education and to facilitate further interventions for students with unhealthy digitally mental behavior.

Through a concept analysis, the five defining attributes of digital resilience were characterized: understanding online threats, knowing solutions, learning knowledge and skills, recovering from stress, and moving forward through self-efficacy. They are influenced by personal internal and external factors when facing the risks of digital technologies. Individuals who have digital resilience by the above-mentioned attributes could be conducive to their psychosocial and behavioral performance and functioning.

Earlier digital resilience research focused mainly on cybersafety or technological support and mostly within the context of a technical perspective, organizational perspective, and national/societal perspective (9, 14, 17). Our work contributed to this gap in the literature by focusing on an individual perspective. The emergence of significant risk factors in the digital world means that people experience distress during their use of digital technologies and perceive them as a challenging threat. The current study made a clear elaboration that threats related to digital technologies are a basic antecedent of resilience. Considering the constantly changing digital landscape and the unpredictable and evolving nature of digital risks, it is difficult to avoid online risks or to prepare a person for all possible online risks. However, digital resilience can be observed in individuals’ ability to overcome exposure to many adversities or threats such as excessive use of online technology (16, 32) and digital burnout or failure (16, 46). Further, digital resilience can play a major role in promoting sustainable, productive, engaging, and healthy learning for students (10, 21, 35) and has a positive influence on self-efficacy (13, 34). When digital resiliency attributes are absent, some negative outcomes may occur such as intense anxiety, fear, or feelings of distress (16, 27).

Developing effective intervention strategies that target digital resilience is essential. However, the way to develop digital resilience in students has been overlooked. Although some studies have contributed practical implications regarding how school educational institutions facilitated resilience in face of the COVID-19 pandemic lockdown, there are no studies that have specifically focused on the interventions for enhancing digital resilience. In this paper, we would offer some key recommendations for the application of the concept in future research. First, we should promote awareness that digital resilience grows through online use and learned experience and cannot be developed through the avoidance of the digital world. Hence, it is especially important to harness the digital resilience of individuals (48). Second, it is necessary to examine the conceptualization of digital resilience in school education settings to further construct an operational definition by mixed research. It is worth noting that cultural context should be recognized as an important factor influencing individual digital resilience (30, 34) and should therefore be considered in the process of concept examination. Third, strategies should be taken to reconstruct cognitive responsibilities like cognitive training that focuses on creating attitudinal change in an effort to influence online behavior. In this process, educators should help subjects feel responsible for their actions and be able to control their behaviors in the face of online threats (19). Additionally, the preliminary conceptual model of digital resilience needs to be further finalized through qualitative studies, which may be later used as a guide for exploring resilience-based interventions for vulnerable students.

Our findings are in accordance with the current trend that resilience is a dynamic, modifiable process (1, 2). We believe that digital resilience is the product of a dynamic process between an individual (e.g., personality) and their environment (e.g., social support). As Cliff Manning mentioned, digital resilience is a dynamic personality asset that grows from digital activation, i.e., through engaging with appropriate opportunities and challenges online, rather than through avoidance and safety behaviors (49). This shows that the extent to which someone can be resilient will fluctuate over time and is intensively influenced by the context. Longitudinal study designs may be taken into consideration to determine the timing and frequency of assessment on digital resilience.

Several limitations of this study need to be acknowledged. First, in the selection process of included studies, the study is not a systematic review or meta-analysis. Consequently, no quality assessment of included studies or statistical analyses were performed. Thus, there might be a risk of bias in results using a text description. Because of this, we need to be careful regarding utilizing the results. Second, since we only analyzed digital resilience in an English context, there might be a risk of bias in language as only English-written articles were included in the analysis. Critical articles may have been missed that were not available in English. Third, within our search strategy, we only included articles that specifically mentioned the concept of digital resilience. This might be the failure to cover all possible components of digital resilience. Some redundant attributes may overlap the antecedents or consequences. Considering this limitation, only five defining attributes were identified in this study because they provided a broad insight into the concept of digital resilience in school education as a response to online technology-related threats. In addition, most of the included studies came from Europe, Asia, and North America, as we did not find enough digital resilience studies from most developing countries. Therefore, studying digital in different social contexts is also necessary because socioeconomic conditions can greatly influence the psychosocial behaviors of people.

This study is the first step in a broader research protocol to enhance individual digital resilience in school education settings. Digital resilience as a fresh and alien concept in school education is best used *via* a qualitative analysis first. Because there are no standard or matching measurements, we will develop a battery of instruments in the next phase, which is beneficial to evaluate and identify vulnerable individuals in low digital resilience. To enhance digital resilience in school education, future interprofessional curricula will be formulated to provide for students with the struggle of their own digital technology-related threats. It is time to conduct proper training on students who may be exposed to different types of risks within different contexts of online activities. These findings can, in turn, inform schools and families about the applicability of resilience-based interventions and the opportunities to guide individuals to positive adaptation. Overall, it is not possible to fully guard against or eliminate all digital threats but with digital resilience, individuals are competent to resist digital challenges or threats.

## Conclusion

This analysis proposes a new conceptualization of individual digital resilience in higher education for the first time based on the identified defining attributes, antecedents, and consequences. We described digital resilience as a circular process toward greater wellbeing in the form of behavioral performance and psychosocial functions when faced with threats, challenges, or adversity during the use of technologies. The presence of resiliency attributes such as understanding online threats, knowing solutions, learning knowledge and skills, recovering from stress, and moving forward through self-efficacy enhances the capacity to be digitally resilient and maintain health promotion. The concept clarification is important for educational work. This concept analysis provides valuable insights that can be used for developing digital resilience intervention measures that may facilitate an early recognition of students’ need for psychosocial support within the context of school education.

## Data Availability Statement

The original contributions presented in the study are included in the article/supplementary material, further inquiries can be directed to the corresponding author/s.

## Author Contributions

HS and CY: conceptualization. HS, QQ, and SH: data acquisition and analysis. HS, CY, SH, and QL: manuscript preparation and modification. All authors contributed to the article and approved the published version of the manuscript.

## Conflict of Interest

The authors declare that the research was conducted in the absence of any commercial or financial relationships that could be construed as a potential conflict of interest.

## Publisher’s Note

All claims expressed in this article are solely those of the authors and do not necessarily represent those of their affiliated organizations, or those of the publisher, the editors and the reviewers. Any product that may be evaluated in this article, or claim that may be made by its manufacturer, is not guaranteed or endorsed by the publisher.
